# Supplementary material for: IL‐23 drives differentiation of peripheral γδ17 T cells from adult bone marrow‐derived precursors
Source: EMBO Rep. 2017 Aug 30;18(11):1957–67. doi: 10.15252/embr.201744200 (PMC5666615; doi:10.15252/embr.201744200)
Supplement: Supplementary file 1 — Expanded View Figures PDF [file EMBR-18-1957-s001.pdf]

Expanded View Figures

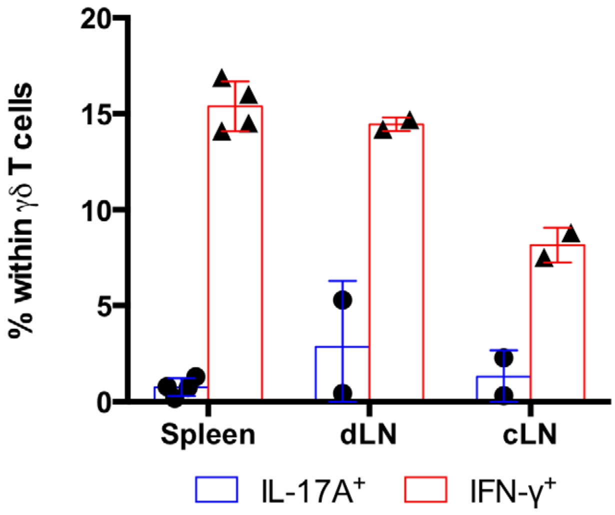

Figure EV1. Bone marrow chimeras contain IFN-γ^+ but not IL-17$^+$ γδ T cells.

Flow cytometry analysis of intracellular IL-17A (blue bars) or IFN-γ (red bars) expression among gated Thy1.1$^+$CD3$^+$TCRδ$^+$ cells after stimulation with PMA and ionomycin. Each symbol represents one Thy1.1:TCRδ$^{-/-}$ bone marrow chimera (BMC), and error bars represent mean ± SD.

 © 2017 The Authors

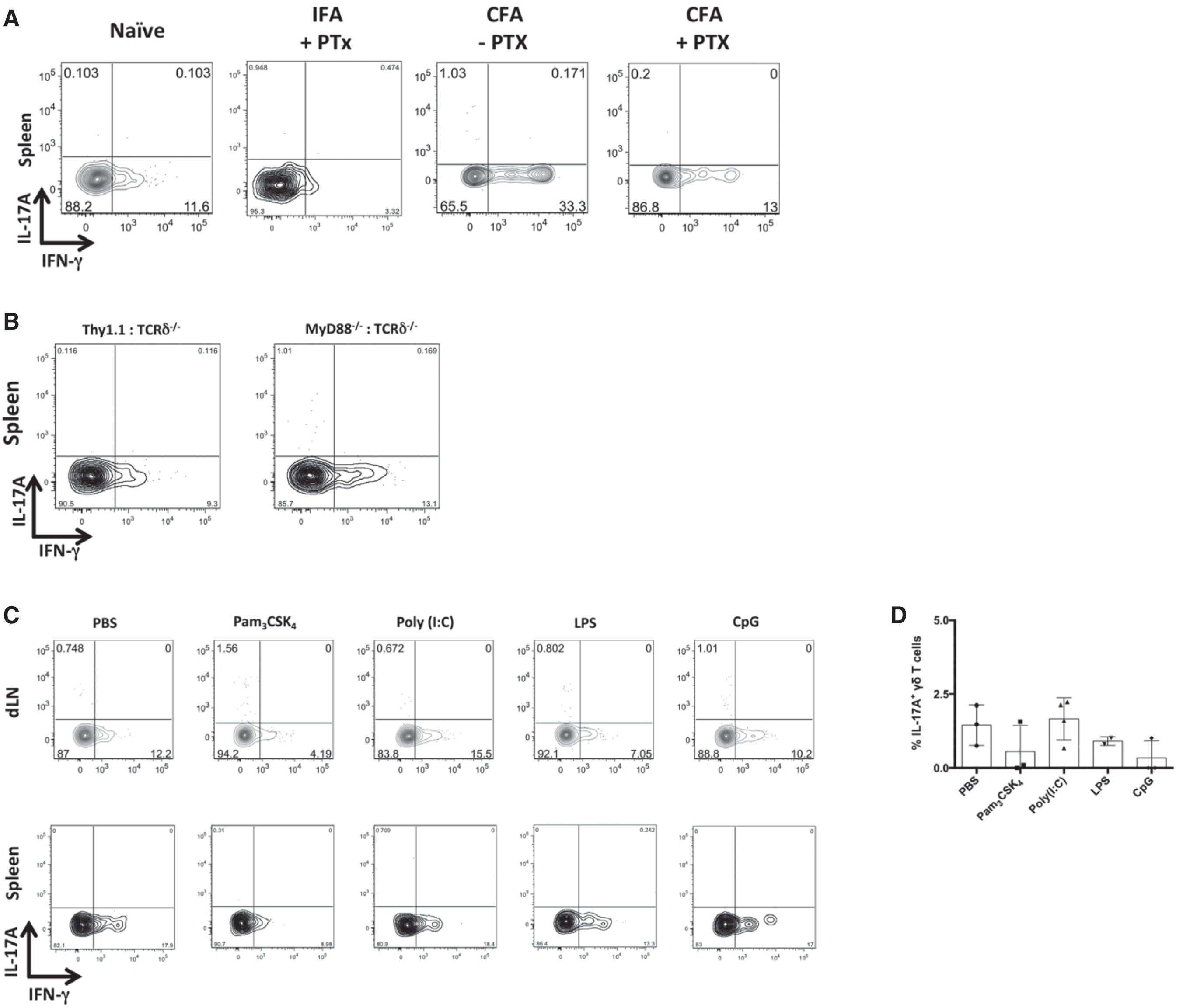

Figure EV2. Induction of IL-17 expression in peripheral γδ T cells does not occur in the spleen and is independent on cell-intrinsic TLR signaling.

A Flow cytometry analysis of intracellular IL-17A and IFN-γ expression in Thy1.1$^+$CD3$^+$TCRδ$^+$ cells isolated from the dLN of Thy1.1:TCRδ$^{-/-}$ BMCs injected subcutaneously with IFA or CFA followed or not by PTx administration.

B Flow cytometry analysis of intracellular IL-17A and IFN-γ expression in Thy1.1$^+$CD3$^+$TCRδ$^+$ cells isolated at day 7 p.i. from the spleens of Thy1.1:TCRδ$^{-/-}$ or MyD88$^{-/-}$:TCRδ$^{-/-}$ BMCs immunized subcutaneously with CFA and given 200 ng of PTx i.v. on days 0 and 2 p.i.

C, D Flow cytometry analysis and frequencies of intracellular IL-17A and IFN-γ expression in Thy1.1$^+$CD3$^+$TCRδ$^+$ cells isolated at day 3 p.i. from the dLN and spleens of Thy1.1:TCRδ$^{-/-}$ BMCs injected subcutaneously 50 μg of each individual TLR agonist (Pam$_3$CSK$_4$, Poly(I:C), LPS or CpG).

Data information: (A–D) Data pooled from two independent experiments. Each symbol represents one individual BMC. Error bars represent mean ± SD.

© 2017 The Authors

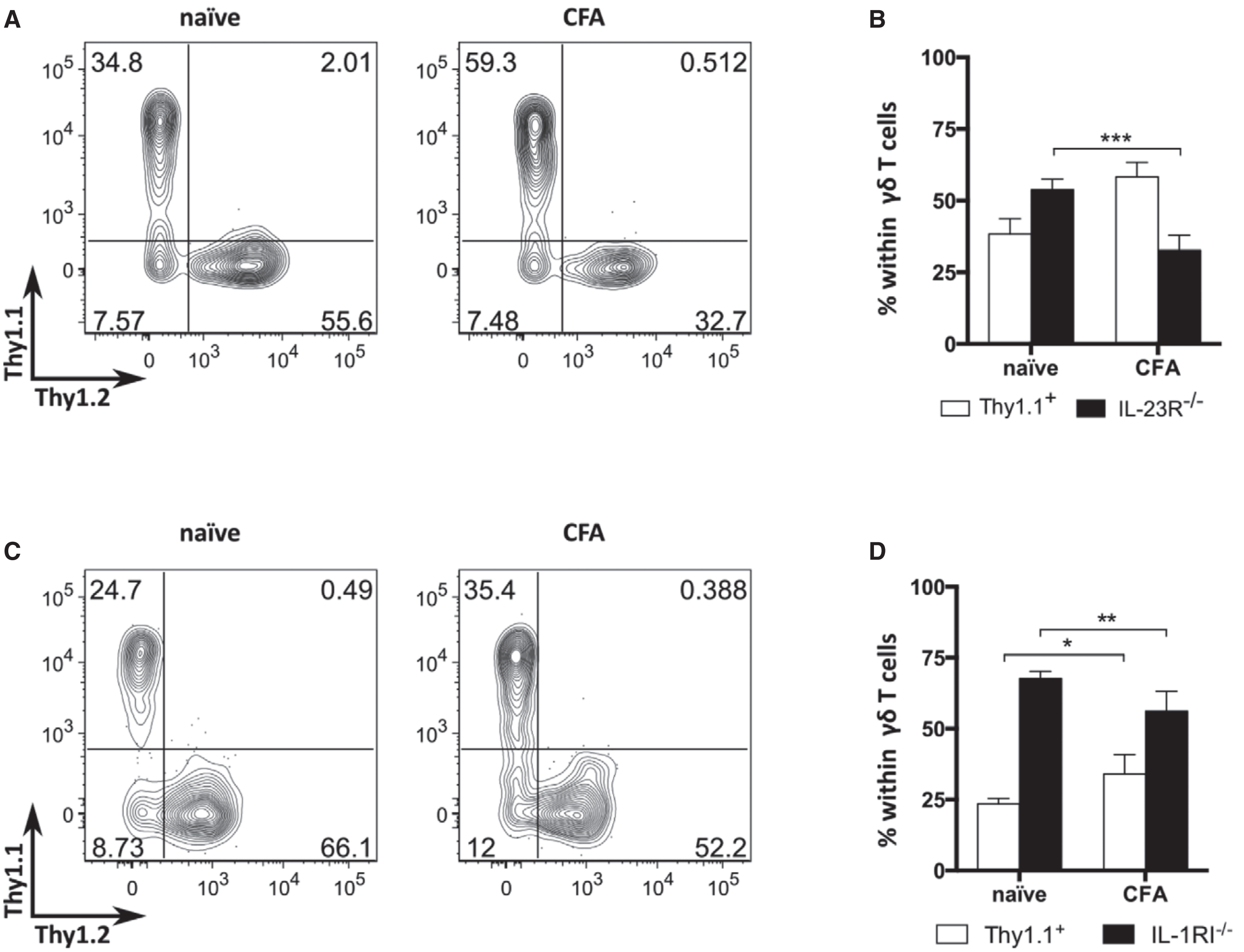

Figure EV3. γδ17 T-cell composition in naïve versus immunized mixed BMCs.

WT ($Thy1.1^+$) and $IL\text{-}23R^{-/-}$ ($Thy1.2^+$) or $IL\text{-}1RI^{-/-}$ ($Thy1.2^+$) bone marrow total cells were mixed at 1:1 ratio to reconstitute lethally irradiated $TCR\delta^{-/-}$ hosts. After 8 weeks, mice were injected subcutaneously with CFA and given 200 ng of PTx i.v. on days 0 and 2 p.i. for additional adjuvant effect. "Naïve" refers to non-immunized controls.

- A Flow cytometry analysis of $IL\text{-}23R^{+/+}$ ($Thy1.1^+Thy1.2^-$) and $IL\text{-}23R^{-/-}$ ($Thy1.1^-Thy1.2^+$) within total $CD3^+TCR\delta^+$ cells. Data are representative of two independent experiments.
- B Frequencies of $IL\text{-}23R^{+/+}$ ($Thy1.1^+Thy1.2^-$; white bar) and $IL.23R^{-/-}$ ($Thy1.1^-Thy1.2^+$; black bar) within $CD3^+TCR\delta^+$ cells from the dLN of naïve or CFA-immunized BMCs.
- C Flow cytometry analysis of $IL\text{-}1RI^{+/+}$ ($Thy1.1^+Thy1.2^-$) and $IL\text{-}1RI^{-/-}$ ($Thy1.1^-Thy1.2^+$) within total $CD3^+TCR\delta^+$ cells. Data are representative of two independent experiments.
- D Frequencies of $IL\text{-}231^{+/+}$ ($Thy1.1^+Thy1.2^-$; white bar) and $IL.1RI^{-/-}$ ($Thy1.1^-Thy1.2^+$; black bar) within $CD3^+TCR\delta^+$ cells from the dLN of naïve or CFA-immunized BMCs.

Data information: (A, B) Data pooled from two independent experiments (n = 3–8 mice per group). (C, D) n = 4–5 mice per group. (B, D) Error bars represent mean ± SD. $^*P < 0.05$; $^{**}P < 0.01$; $^{***}P < 0.001$ (Mann–Whitney U-test).

 © 2017 The Authors

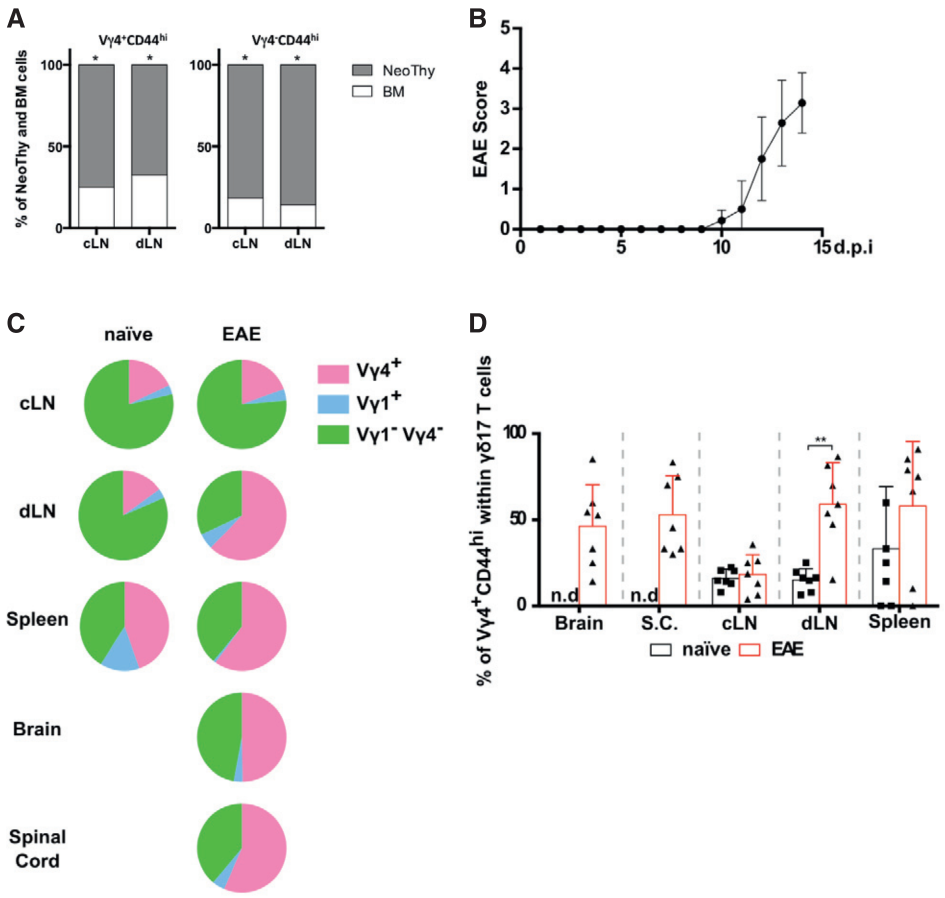

Figure EV4. γδ17 T cells in naïve NeoThy + BM chimeras are mostly of neonatal thymic origin.

A Flow cytometry analysis of the fraction of $Thy1.1^{+}Thy1.2^{-}$ (BM; white) or $Thy1.1^{+}Thy1.2^{+}$ (NeoThy; gray) cells among $V\gamma4^{+}$ (left) or $V\gamma4^{-}$ (right) subsets of $CD3^{+}TCR\delta^{+}IL\text{-}17A^{+}$ lymph node cells from lethally irradiated mice transplanted with both neonatal thymocytes and bone marrow (n = 7 mice). *$P < 0.05$ (Student's t-test). Data are representative of two independent experiments.

B Mice were observed daily and scored for clinical signs of EAE.

C Pie chart distribution of TCR-Vγ chain usage of γδ17 T cells from different organs of naïve (left) or EAE-immunized (right) NeoThy+BM chimeras, as determined by flow cytometry analysis of TCR-Vγ1 and TCR-Vγ4 expression within the $CD3^{+}TCR\delta^{+}IL\text{-}17A^{+}$ population.

D Frequencies of $V\gamma4^{+}CD44^{hi}$ cells within the $CD3^{+}TCR\delta^{+}IL\text{-}17A^{+}$ population determined by flow cytometry in different organs of naïve (black bar) or EAE-immunized (red bar) NeoThy + BM chimeras.

Data information: (A–D) Data pooled from two independent experiments. (D) Error bars represent mean ± SD. **$P < 0.01$; (Mann–Whitney U-test).
